# Tremendous advances, multifaceted challenges and feasible future prospects of biodegradable medical polymer materials

**DOI:** 10.1039/d4ra00075g

**Published:** 2024-10-11

**Authors:** Fulong Li, Chao Chen, Xiaohong Chen

**Affiliations:** a School of Materials & Chemistry, University of Shanghai for Science & Technology Shanghai 200093 China 15737319783@163.com chaochen0612@163.com xiaohongchen_ust@163.com +86 15737319783 +86 17626650845 +86 13167086410; b Shanghai Engineering Technology Research Center for High-Performance Medical Device Materials Shanghai 200093 China

## Abstract

In recent years, biodegradable medical polymer materials (BMPMs) have stood out among many biomedical materials due to their unique advantages, such as high mechanical strength, good biocompatibility, strong corrosion resistance and excellent processability. In this review, we first provide a brief introduction of biodegradable medical materials from both natural and synthetic perspectives, and then systematically categorize BMPMs based on their applications in clinical medicine and highlight the great progress they have made in recent years. Additionally, we also point out several overlooked areas in the research of BMPMs, offering guidance for comprehensive future exploration of these materials. Finally, in view of the complex challenges faced by BMPMs today, their future directions are scientifically proposed. This work contributes to the ongoing efforts of BMPMs in the biomedical field and provides a steppingstone for developing more effective BMPM-based products for clinical applications.

## Abstract 2

This work contributes to the ongoing efforts of BMPMs in the biomedical field and provides a steppingstone for developing more effective BMPM-based products for clinical applications.

## Graphical abstract

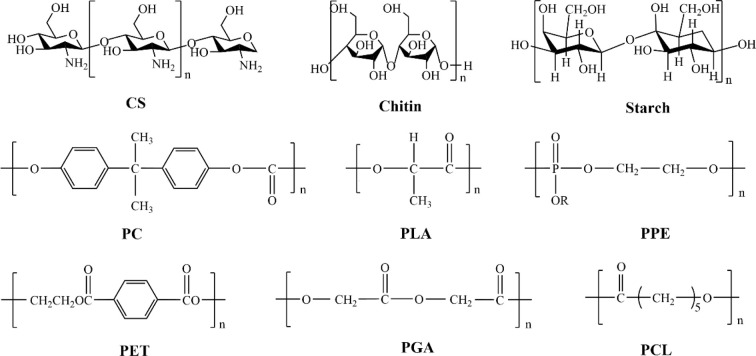

## 1. Introduction

Medical polymer materials can be used for clinical diagnosis, disease treatment, surgical repair, and even human tissue and organ replacement.^1–5^ Based on whether the materials themselves are biodegradable, they tend to be broadly divided into two categories. Materials that can be hydrolyzed or enzymatically degraded into low molecular weight compounds or monomers within the biological system are defined as biodegradable medical polymer materials (BMPMs).^6^ Conversely, if materials cannot be hydrolyzed or enzymatically degraded, they are referred to as non-BMPMs. Clearly, BMPMs are a type of semi-automated functional material, as BMPMs can degrade automatically after service without the need for additional manual intervention.^7^ More importantly, extensive research has shown that the molecular structure, chemical composition, and physical properties of BMPMs are extremely similar to those of biological tissues. When human tissues need to be transplanted due to disease or trauma, it is difficult to satisfy the demand for autologous organs, except for a small amount of skin. Alternative measures such as allografts or xenografts often result in strong rejection reactions,^8,9^ which in severe cases directly induces surgical failure.^10,11^ In this situation, it is common to consider using BMPMs to repair or replace damaged parts of the organism.^12^ Consequently, BMPMs occupy an irreplaceable position in clinical applications.

Unlike general medical materials, BMPMs come into direct contact with human tissues or organs, thereby dominating the lives of patients. Once the performance of BMPMs is inaccurate to the actual needs of the human body, it can pose a serious threat to patient safety. Therefore, some stringent requirements for BMPMs are clearly listed in Table 1. Only BMPMs with the following properties can be considered as the ideal materials.

**Table 1:** The basic performance requirements of BMPMs in clinical application

Performance requirements	Objectives	Ref.
Good biocompatibility	To avoid the rejection reaction	13 and 14
Steady mechanical properties	To ensure that the material will serve for a long time	15–17
Credible biosecurity	To make sure to be harmless to human health	18 and 19
Appropriate degradation rate	To match perfectly with the rate of tissue regeneration	20 and 21
Excellent processability	To process into complex structures as required	22 and 23
Outstanding bioactivity	To induce specific biochemical reactions between the material and the surrounding host tissue	24

**Fig. 1:**
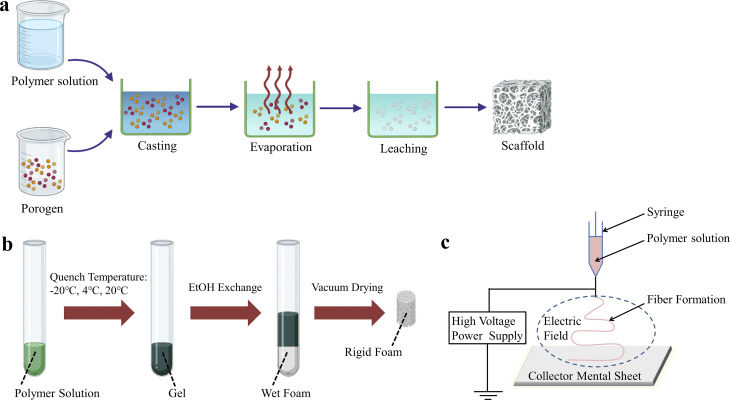
Schematic representation of some conventional techniques for the preparation of BMPM-based products. (a) SCPL. (b) TIPS. (c) Electrospinning.

So far, the techniques used to prepare BMPM-based products in biomedical field have been widely reported, mainly including solvent casting/particle leaching (SCPL), thermally induced phase separation (TIPS), electrospinning, and three-dimensional (3D) printing. Among them, the first three are traditional preparation methods (Fig. 1). Although these methods are still used in biomedicine due to their mature technology, stable process, and the ability to maintain the original chemical properties of the materials, they also have some shortcomings, such as long production cycles, high energy consumption, low mechanical strength, and the inability to achieve complex personalization. In recent years, with continuous advancements in technology, 3D printing technology has shown promising applications in biomedicine and has gained widespread attention from researchers.

**Fig. 2:**
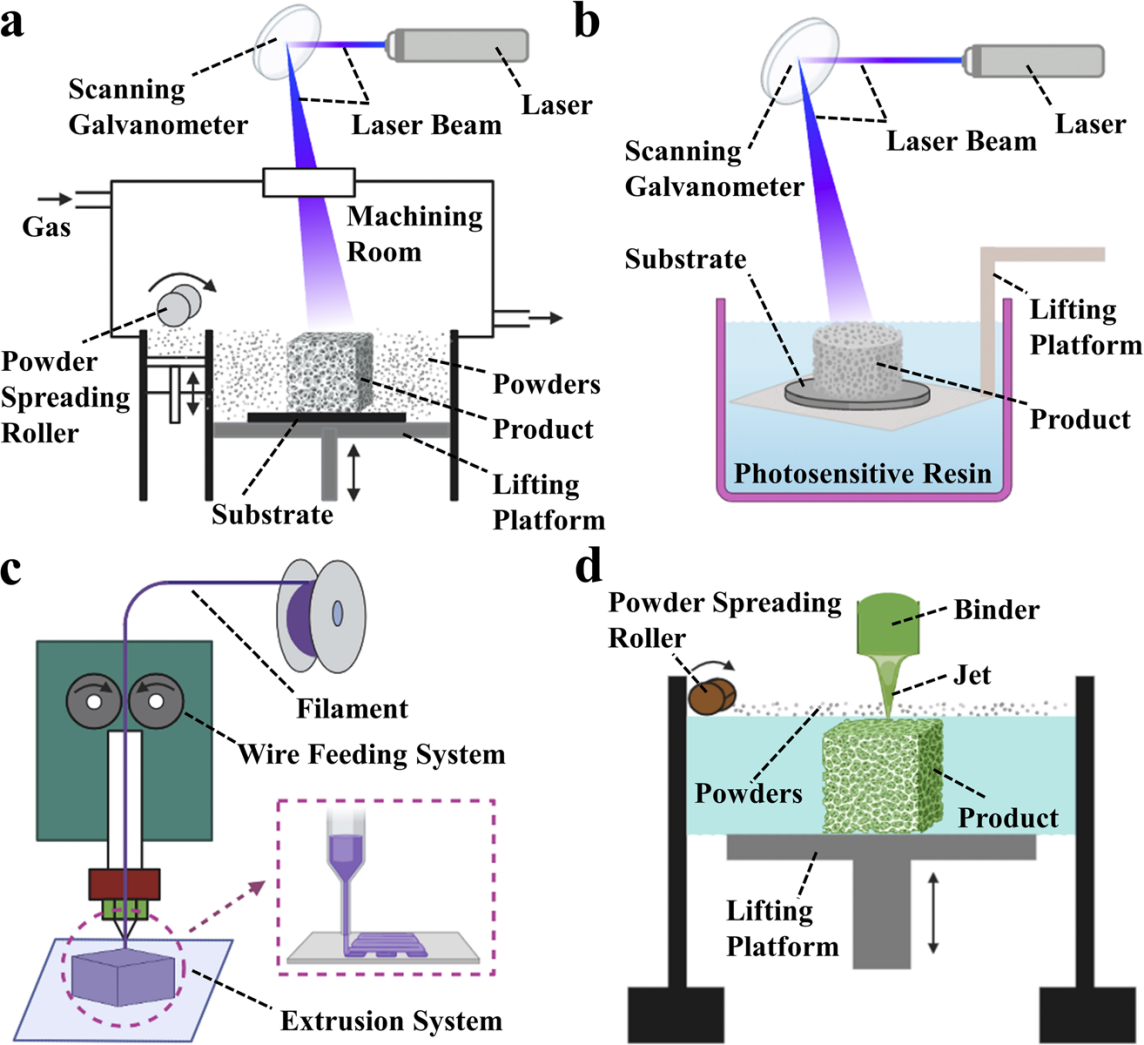
Schematic diagram of 3D printing technology. (a) Stereo lithography apparatus (SLA). (b) Selective laser sintering (SLS). (c) Fused deposition modeling (FDM). (d) 3D printing (3DP).

3D printing technology, also known as additive manufacturing (AM) or rapid prototyping (RP),^25–30^ is one of the most common techniques for processing materials into products.^31–33^ It utilizes energy sources such as light, electricity, and heat to melt, sinter, or jet materials, stacking them layer by layer to form predetermined 3D scaffold structures. What makes 3D printing technology unique compared to traditional techniques is that it allows for the rapid manufacture of solid parts based on computer-drawn file graphics without the need for molds^34–37^ thus greatly reducing production costs and cycle times.^38–40^ The greatest advantage of 3D printing is its ability to create personalized designs based on the patient's medical images, including hollow structures and complex geometries. Additionally, customized pore sizes and excellent mechanical properties are notable features of 3D printed bone scaffolds. Currently, the primary 3D printing processes used to fabricate bone tissue scaffolds include selective laser sintering (SLS), stereolithography (SLA), fused deposition modeling (FDM) and 3D printing (3DP), with corresponding schematic diagrams shown in Fig. 2.

**Fig. 3:**
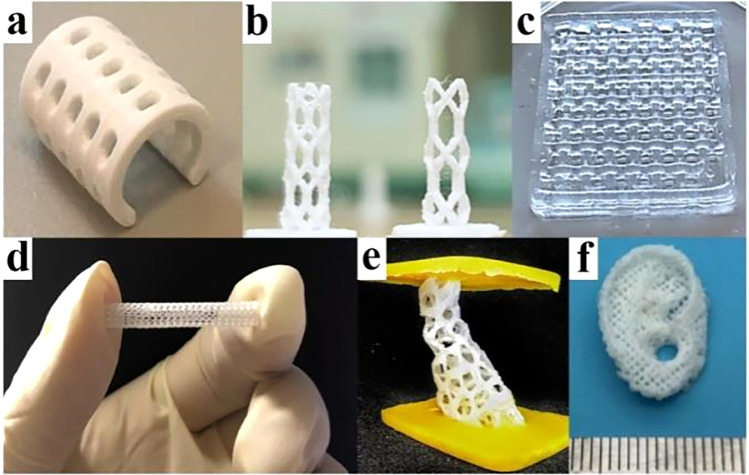
A few products by 3D printing technology. (a) PCL splint. Reproduced from ref. 44 with permission from Elsevier, copyright 2021. (b) Coronary artery stent. Reproduced from ref. 45 with permission from Elsevier, copyright 2021. (c) Polyvinyl alcohol and cellulose nanofibril hydrogel. Reproduced from ref. 46 with permission from Elsevier, copyright 2021. (d) Vascular stent. Reproduced from ref. 47 with permission from Elsevier, copyright 2020. (e) Bone scaffold. Reproduced from ref. 48 with permission from Elsevier, copyright 2021. (f) Auricular scaffold. Reproduced from ref. 49 with permission from Elsevier, copyright 2021.

More surprisingly, a large number of studies have shown that the combination of BMPMs and 3D printing technology can not only enable personalized design, but also give the product its own outstanding performance advantages without any reservations.^41–43^Fig. 3 shows some of the biomedical products obtained by 3D printing technology, which have shown excellent performance after continuous testing by designers.

The objective demands in the medical field have driven the development of BMPMs. In recent years, with the increasing clinical emphasis on the requirements of minimal invasion and high precision, BMPMs have been gradually pushed to the forefront, and their unique advantages have been increasingly recognized, explored and utilized. This review systematically categorizes BMPMs according to their biomedical applications and pays great attention to the significant advances made in the medical field over the past few years. Finally, considering the numerous challenges currently faced, the future directions of BMPMs are rationally proposed.

## 2. BMPMs

**Fig. 4:**
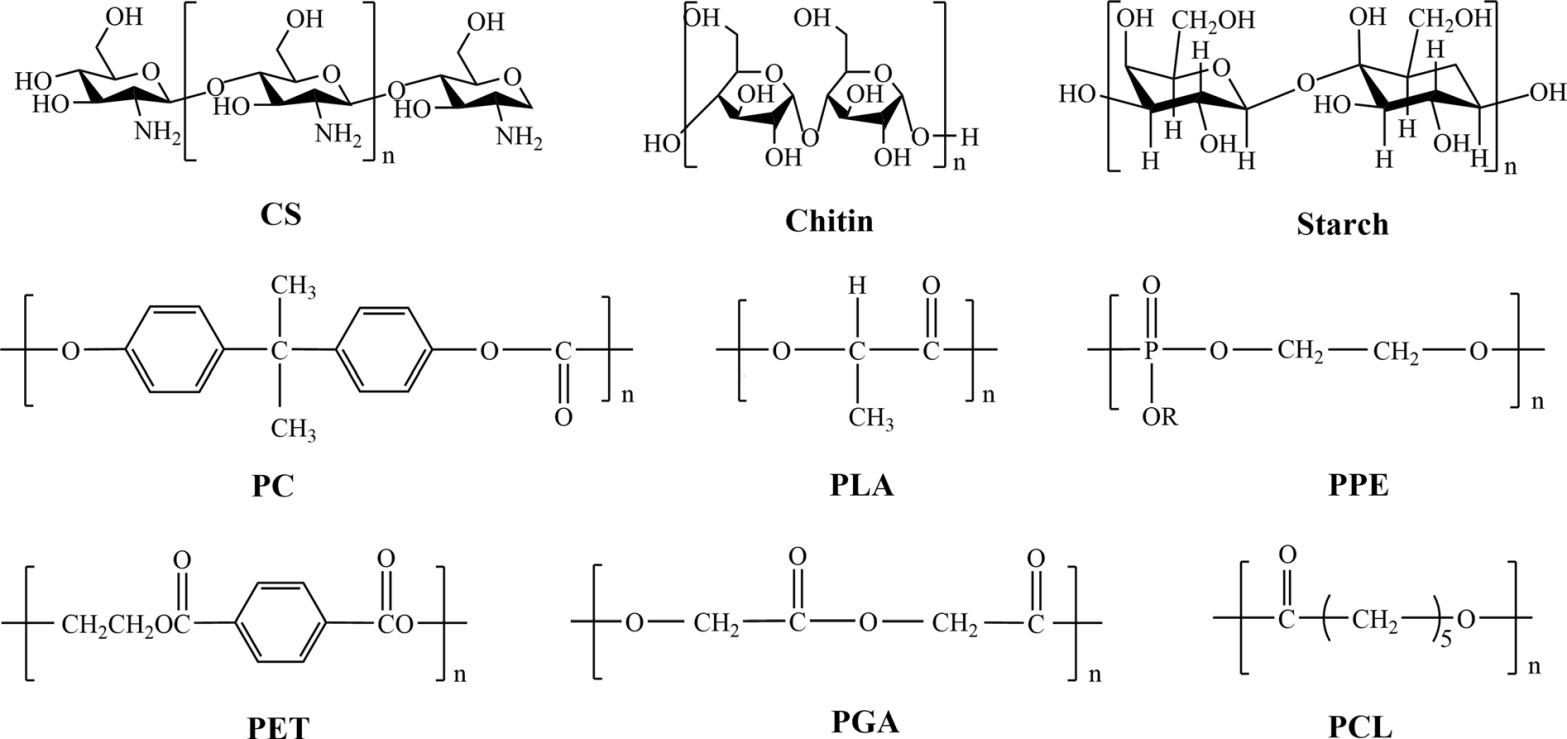
Chemical structures of all the aforementioned BMPMs.

There are two main sources of BMPMs.^50,51^ One is in the form of natural products from the biological community, which are biocompatible and non-toxic,^52^ including chitosan (CS), chitin and starch. The other type is obtained through synthesis and mainly involves polylactic acid (PLA), polycarbonate (PC), polyphosphoric ester (PPE), polycaprolactone (PCL), polyglycolic acid (PGA), and polyethylene terephthalate (PET). Fig. 4 illustrates the chemical structures of these materials. As the former need to be isolated, refined and purified before use, resulting in low purity.^53^ Unfortunately, they usually exhibit short-term degradation and inadequate mechanical properties.^54^ Therefore, they are highly limited in practical applications. In contrast, the latter offer more options.^55^ The most notable advantage of synthetic materials is their controllability in terms of mechanical strength, hydrophilicity–hydrophobicity and degradation rate. Additionally, synthetic materials are characterized by high mechanical properties, good thermal stability, and resistance to biological aging, making them widely applicable in artificial organs (such as kidneys, hearts, and lungs), artificial tubes (blood vessels, esophagus, intestines, and urethra), as well as vascular scaffolds.^56^ Therefore, in this paper, synthetic BMPMs were chosen as the focus of discussion.

**Fig. 5:**
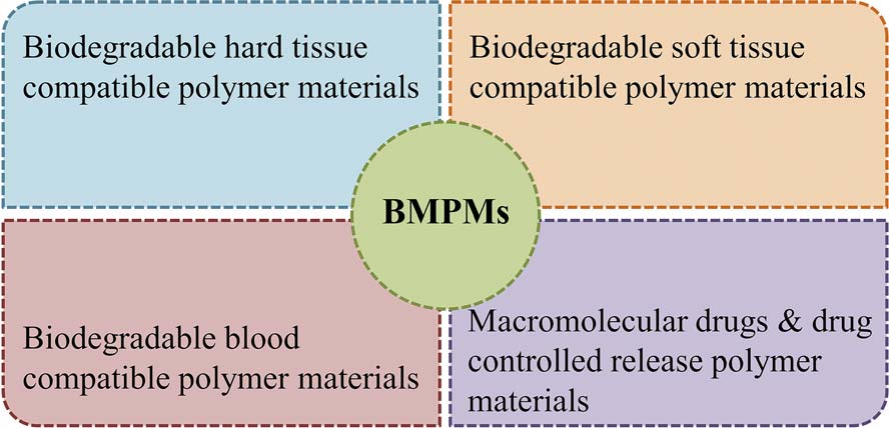
Classification results of BMPMs according to medical applications.

To date, a number of BMPMs with excellent performance in the medical field have been reported. However, authorities in relevant academic fields have not yet developed a set of well-recognized and unified classification criteria. In this review, BMPMs are systematically categorized by medical applications, and the results are shown in Fig. 5. It is worth emphasizing that this classification method is not exclusive, as some materials are widely used in different applications due to their outstanding properties.

### 2.1. Biodegradable hard tissue compatible polymer materials

A class of synthetic degradable polymers capable of undertaking the task of protecting vital organs, shaping, supporting body weight and achieving intra-biological movement are defined as degradable hard histocompatible polymers,^57,58^ such as polylactic acid, PC, PCL, PGA and PC. These materials are widely used as artificial bone substitute materials because of their light specific gravity, good biocompatibility, high mechanical properties, and low corrosiveness. When the human body has serious bone defects or pathological changes caused by viral infection, tumor removal, traumatic injury, congenital dysplasia, *etc.*, artificial bone can be used to fill and repair the bone and eventually restore its original function.^59^

Among the aforementioned materials, PLA is a typical representative of biodegradable hard tissue compatible polymeric materials.^60^ It is a non-toxic polymer compound formed from the polymerization of lactic acid, free of reactive functional groups and hydrophilic groups, with good biocompatibility, adjustable degradability and excellent physical properties.^61–67^ The greatest advantage of PLA is that it can be completely degraded *in vivo* and eventually generate CO_2_ and H_2_O.^68^ Apart from this, no other degradation products are generated.^69^ Therefore, PLA is unanimously recognized as the most desirable and promising artificial bone replacement material^70–73^ and is highly valued.

The most concerning issues regarding PLA after implantation are its mechanical properties, biocompatibility, cytotoxicity, and degradability. Good mechanical properties are fundamental for the successful implantation of artificial bones, ensuring that the scaffold can provide long-term support. Excellent biocompatibility is the key for the successful integration of the artificial bone with the biological system, preventing inflammation or rejection reactions. The cytotoxicity of PLA in the body determines its suitability for biological use. The degradation process is crucial for bone replacement and regeneration, with the ideal scenario being that the degradation rate of the material aligns with the rate of new bone regeneration. If the former occurs earlier than the latter, it will undoubtedly lead to surgical failure. Conversely, an undegraded bone scaffold can prevent the formation of new bone, resulting in deformities.

**Fig. 6:**
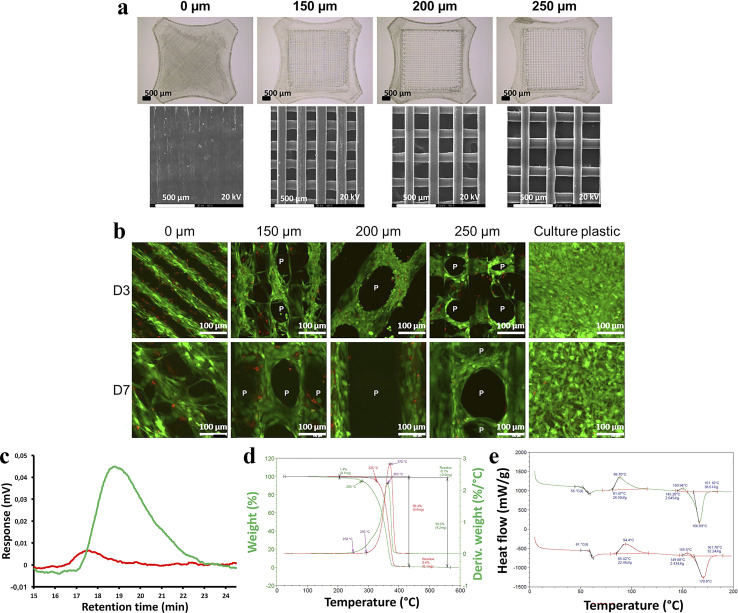
(a) Scaffolds with 0, 150, 200, 250 µm pore size by binocular microscopy and scanning electron microscopy. (b) Size exclusion chromatographic assay of printed PLA scaffolds. (c) Thermogravimetric analysis of printed PLA scaffolds. (d) Differential scanning calorimetric assay of printed PLA scaffolds. (e) HBMSC colonization of sterilized PLA scaffolds was evaluated after 3 and 7 days of culture using fluorescent microscopy after live/dead staining (green/red) (*n* = 3). Reproduced from ref. 74 with permission from Wiley, copyright 2018.

Gremare *et al.*^74^ fabricated three PLA scaffolds with pore sizes of 150, 200 and 250 µm by the FDM technique. The survival rates of human bone marrow stromal cells (HBMSC, live/dead status indicated by green/red fluorescence) cultured on the three PLA scaffolds for 7 days showed that HBMSC were uniformly distributed throughout the grid, demonstrating high viability regardless of pore size. These results provide strong evidence that PLA printed scaffolds possess good cell compatibility and non-toxicity. Additionally, the authors found that PLA-printed scaffolds exhibited lower pore size and larger diameter than expected. More interestingly, they also discovered that the 3D printing process reduced the molecular weight and degradation temperature of PLA, but these unexpected findings do not negate the fact that PLA printed scaffolds provide personalized treatment for patients with bone defects (Fig. 6).

However, numerous experimental results have shown that pure PLA as a bone repair material typically exhibits varying degrees of deficiencies in mechanical properties. Therefore, PLA is usually designed to be blended with reinforcement materials to become a satisfactory product.^75–77^ Considering that pure PLA cannot provide effective support in orthopedic fixation devices due to its poor mechanical strength, Wan *et al.*^78^ proposed the preparation of PLA/bones/polybutylene succinate (PLA/PBSA) biocomposites using pork bone powder and PBSA as toughening agents. The mechanical strength of PLA/bones/PBSA biocomposites increased with the increase of the content of PBSA and pork bone, reaching maximum values when both PBSA and pig bone contents are 10 wt%, with tensile strength of 48.5 ± 0.2 MPa, flexural strength of 79.1 ± 0.1 MPa, and notched impact strength of 15.8 ± 0.3 kJ m^−2^. In addition, the authors also observed through *in vivo* bone repair experiments that the bone repair at the damaged site of rabbits was nearly completed within four months after filling with 80 wt% PLA/10 wt% bones/10 wt% PBSA.

Wang *et al.*^79^ fabricated PLA/nanohydroxyapatite (n-HA, 50 nm to 80 nm in diameter) composite scaffolds (Pn scaffolds) by the FDM technique. When the specific gravity of n-HA reached 40%, the scaffold could not maintain its intact shape after the compression test. A reasonable explanation can be derived from SEM images: the flatness of the scaffold surface was gradually transformed from smooth to rough as the amount of n-HA increased, which led to a significant decrease in compressive strength. However, the osteogenic effect of the Pn composite scaffold was optimistic. The Pn30 scaffolds in rabbit femur fitted closely to the bone defect site, with regenerated bone tissue attaching to the scaffold surface and growing along the pores within three months. The number of new bone layers gradually increased, ultimately closing the bone defect. In contrast, in the Pn0 group, the new bone was sporadically distributed. The Pn composite scaffold provides an excellent strategy for the repair of large bone defects.

In addition, several researchers have selectively mixed several biodegradable metals or their alloys with PLA to obtain bone substitute materials with high mechanical strength. For example, the biodegradable Mg/PLA composite bone plates designed by Rizvi *et al.*,^80^ Mehboob *et al.*,^81^ Ali *et al.*,^82^ as well as the PLA/316L composite scaffold proposed by Jiang *et al.*^83^ also exhibited impressive mechanical properties, biodegradability, and cell safety. They successfully revealed the potential applications of PLA in hard tissues. However, a detailed discussion of these studies is not provided here.

Similarly, in addition to PLA, other biodegradable hard tissue compatible polymer materials have also demonstrated excellent clinical applications. For example, in the study by Chen *et al.*,^84^ the authors used 3D printing technology to fabricate a porous biodegradable HA/CMCS/PDA scaffold using hydroxyapatite (HA), carboxymethyl chitosan (CMCS), and polydopamine (PDA). They found that the degradation of HA/CMCS/PDA scaffolds may lead to a significant decrease in compressive strength and compressive modulus. After implanting the scaffold into defective rabbit femurs, cortical bone formed within 12 weeks, while the scaffold was almost completely degraded. In contrast, the blank control group still had significant bone defects.

Throughout the above examples, biodegradable hard tissue polymer materials combined with 3D printing technology can produce a variety of bone substitutes. However, these products have not yet been fully recognized. There are two main scientific explanations for this viewpoint. To begin with, the shortage of experimental subjects is a significant barrier to the clinical application of biodegradable hard tissue compatible polymer materials. Currently, most of the subjects involved in experiments are lower animals such as rabbits, rats, pigs and dogs, rather than humans. There are huge differences between lower animals and humans in terms of self-protection awareness and behavioral activities after surgery. Secondly, the impact of 3D printing technology on materials cannot be ignored. Obviously, ref. 84 serves as a representative example.

### 2.2. Biodegradable soft tissue compatible polymer materials

Biodegradable soft tissue compatible polymer materials, whose products mainly include artificial blood vessels, artificial skin, artificial muscles, artificial ligaments, *etc.*, are mainly used for soft tissue replacement and repair. Among them, the artificial skin developed *in vitro* is favored by people at home and abroad because it can be applied to treat diabetic foot skin ulcers, epidermolysis bullosa, smooth purpura, burns and skin defects after trauma surgery. The design of the artificial skin must highly mimic the unique structure of natural skin, including the bilayer cellular structure and extracellular matrix, which can create a microenvironment for skin regeneration and selectively direct cell attachment, migration and proliferation to the injured site. The degradation and biocompatibility of materials during the repair process have attracted a great deal of attention from researchers.

Rodriguez-zapater *et al.*^85^ concluded that the optimal measure for benign tracheobronchial stenosis is stent implantation, so they designed an expanded biodegradable polydioxanone (EP BPS) stent by using a single 3.5 EP BPS braided filament. 22 rabbits were divided into 3 groups with different survival times (30, 60, and 90 days after implantation). In the D30 group (*n* = 7), the radiolabel placed on the tracheal stent was detected in all receptors. In contrast, in the D90 group (*n* = 8), the radiolabel disappeared and all tracheas remained patent, indicating that degradation of the BPS occurred between 30 and 90 days. Furthermore, no granuloma or scaffold re-epithelialization were observed when BPS was not completely degraded. Only imprints of the membrane in the epithelium or inward folding of the epithelium over the incompletely covered membrane were noted. Despite the promising results of BPS, some issues warrant further investigation. Firstly, this study was performed in rabbits rather than humans. The trachea of rabbits does have similarities to the human trachea, but the effect of BPS in human tracheas remains unclear. Secondly, the authors assessed the response of healthy rabbit tracheas rather than diseased ones, which severely weakens the credibility of the experimental findings.

Zhang *et al.*^86^ blended the biodegradable elastomer poly(glycerol sebacate) (PGS) with silk fibroin (SF) to fabricate a series of novel PGS/SF artificial blood vessels by electrospinning. The authors found that sebacic acid produced from PGS degradation lowered the pH of the PBS degradation solution, while the amino acids from SF degradation had little effect on the pH of the PBS solution but mitigated the acidity increase during PGS degradation. Thus, the incorporation of SF greatly ameliorated the problem of excessive PGS degradation, confirming that the PGS/SF electrospun material has the properties of both components. On day 7 after *in vitro* inoculation of human umbilical vein endothelial cells (HUVECs), all groups except for the pure SF material showed extensive cell adhesion on the surface and began to demonstrate endothelialization, suggesting that PGS/SF electrospun artificial blood vessels can promote cell adhesion, growth, and proliferation.

Zhou *et al.*^87^ developed a multifunctional conductive scaffold (PGFP scaffold) with thermosensitive, injectable, self-healing, controllable conductivity and skin adhesion behavior for multimodal tumor/infection damaged skin treatment by cross-linking branched polyglycerol-amino acids, polypyrrole@polydopamine (PPy@PDA) nanoparticles and aldehyde-based F127. Here, the conductivity and photothermal responsive drug release of the scaffold will not be described in detail. Cytotoxicity tests in L929 cells showed an increasing number of viable cells over 1–5 days, indicating that the PGFP scaffold has good cytocompatibility and proliferation, which is beneficial for wound healing. Moreover, within 14 days post-implantation, the PGFP group demonstrated the best wound healing effect, with a closure rate of 97.7% and nearly complete coverage by new skin. In contrast, the wounds in the control group remained scarred and covered with scars. These results strongly suggest that the PGFP scaffold has a higher healing rate, making it an effective tool for treating damaged skin.

**Fig. 7:**
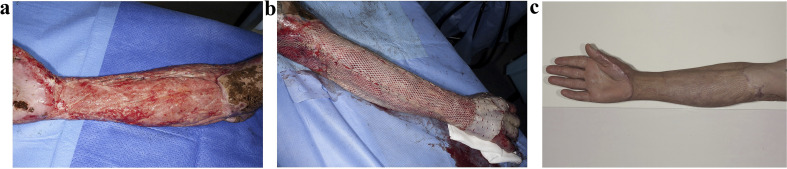
Healing process of a 42 year-old male with 25% flame burn injury treated with BTM. (a) Patchy pink and bright red appearance following delamination of vascularized BTM applied to right forearm at day 28. (b) Meshed split skin grafting. (c) Appearance after using BTM for 12 months. Reproduced from ref. 88 with permission from Elsevier, copyright 2022.

In addition, Lo *et al.*^88^ fabricated a novel polyurethane skin template (NovoSorb^®^ biodegradable template, BTM) to address the issue of insufficient autologous donor skin for early debridement and grafting in a single stage for large-area burns. BTM is a sealing membrane that is bonded together through a polyurethane adhesive layer and biodegradable polyurethane open-cell foam (with a porosity of 90% and pore sizes ranging from 100 to 500 micrometers). Fig. 7 illustrates the process of a burn patient from BTM implantation to wound closure. After BTM incorporation into the wound, the sealing membrane is removed after approximately 28 days, leaving a layered skin graft that can be applied to the new vascularized dermal structures, allowing the wound to close within 12 months. It was clinically demonstrated that no pathological changes or clinical infections were detected during the 12 months follow-up. These results provide sufficient clinical evidence for BTM as an effective skin substitute for treating patients with deep burns.

It is no exaggeration to say that the degradation behavior of degradable soft tissue compatible polymer materials directly determines the success of artificial soft tissue in repairing and regenerating injured areas. Based on the above cases, it is not difficult to find that in the experimental exploration of degradable soft tissue compatible polymer materials, there are few studies on the effects of material degradation processes on soft tissue reconstruction and the dynamic changes in material mechanical behavior. The uppermost reason for this phenomenon is that it is difficult to establish a dual dynamic testing platform, which ultimately leads to the relationship between material degradation and dynamic changes in mechanical behavior becoming unadopted patches. In order to fully grasp the properties of materials and accurately address clinical needs, it is only right that we devote ourselves to it.^89^

### 2.3. Biodegradable blood compatible polymer materials

Biobased biodegradable polymeric materials that are applied in environments involving blood circulation can be defined as biodegradable blood compatible polymeric materials, such as PLA, PCL, PGA, and polyvinyl alcohol (PVA). Due to the specificity of the working conditions and the importance of the contact objects, biodegradable blood compatible polymer materials should not only meet the performance requirements specified for ordinary biomaterials, but also satisfy additional criteria, such as not causing plasma protein denaturation, not affecting the activity of various enzymes, not altering the concentration of electrolytes in the blood, and not provoking harmful immune responses.^90^ Biodegradable vascular scaffolds are one of the most commonly used products in clinical practice,^91^ and they have become the most effective surgical procedure for the treatment of cardiovascular diseases.^92–96^

**Fig. 8:**
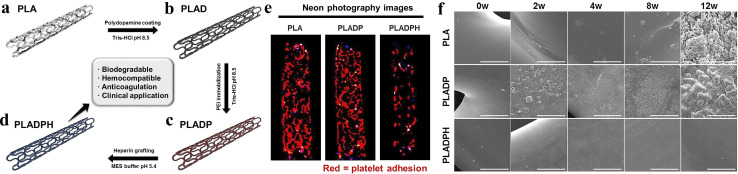
(a) 3D printed bare PLA stent. (b) PDA coating on bare PLA stent. (c) PEI immobilization on PDA coated PLA stent. (d) Hep grafting on PDA/PEI layered PLA stent. (e) Neon photograph images for platelet visualization on PLA, PLADP, and PLADPH (*n* = 3). (f) Surface morphology variation of the PLA, PLADP, and PLADPH throughout the specific time points degraded in PBS solution under pH 7.4 at 37 °C. Reproduced from ref. 97 with permission from Elsevier, copyright 2019.

Lee *et al.*^97^ obtained 3D printed PLA biodegradable polymer scaffolds with high anticoagulation according to the steps shown in Fig. 8. The hemolysis results of PLA, PLADP and PLADPH scaffolds indicated that the presence of heparin significantly reduced the hemolysis rate, with the hemolysis rate of heparinized PLADPH stents being only 1.99 ± 0.81%, making it very suitable for use in the arterial setting. During a 12 weeks degradation period in PBS solution at 37 °C and pH 7.4, the surfaces of both PLA and PLADP stents were accompanied by cracks, pores and even debris, while the PLADPH group exhibited almost no physical changes, which may account for the minimal change in initial weight of the PLADPH stent. This experiment provides a viable option for vascular stents to avoid thrombosis.

**Fig. 9:**
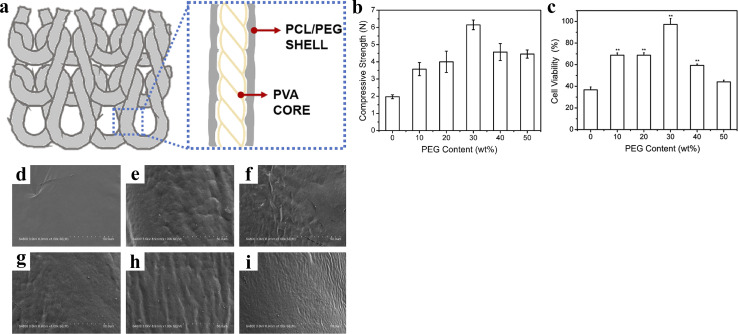
(a) Scheme of composite stent. (b) Compressive strength of composite stents. (c) Cell viability of composite stents (*p < 0.05, **p < 0.01). (d–i) Microstructures of composite stents relative to the PEG content of 0, 10, 20, 30, 40, and 50 wt%. Reproduced from ref. 47 with permission from Elsevier, copyright 2020.

Lin *et al.*^47^ twisted and coated biodegradable PVA yarns with a mixture of PCL/PEG. Then, the coated yarns were braided in the weft direction. After heat treatment, composite scaffolds with a core–shell structure were prepared (Fig. 9). Overall, the mechanical strength of the composite scaffolds first increased and then decreased with the addition of PEG, reaching a maximum of 6.15 N at 30 wt%. Notably, when the PEG content reached this level, the composite scaffolds began to exhibit a wavy surface, which facilitated cell adhesion and proliferation, leading to a cell viability rate of up to 97.32%. However, scaffolds with overly rough surfaces showed a lower cell proliferation rate due to the crowded environment created by a rough matrix and the adverse effects of low nutrients on cell growth. This investigation suggests that the composite structure of bioresorbable vascular scaffolds provides a strong impetus for the development of novel scaffolds.

Subsequently, Lin *et al.*^98^ further proposed a two-layer degradable vascular scaffold model made from PVA yarns with a twist factor of 4. CS coating and genistein (GP) chemical cross-linking were used to secure the interlacing points and enhance the mechanical properties of the braided PVA vascular scaffold, respectively. After testing, the authors found that the stability of the PVA vascular scaffold was greatly improved, with less than 3% weight loss after soaking in PBS solution for 30 days. In addition, CS is positively charged in an acidic environment and can attract negatively charged bacteria such as *Staphylococcus aureus* and *E. coli* (*S. aureus* and *E. coli*), thus affecting the osmotic pressure inside and outside the bacteria. Therefore, the PVA–CS–GP scaffold exerted antibacterial properties. In conclusion, the chemically modified woven PVA vascular scaffold demonstrates satisfactory biological characteristics, making it an ideal structural model for vascular scaffolds in the field of tissue engineering.

The mechanical properties of biodegradable scaffolds play a fatal role in the treatment of cardiovascular diseases. At the same time, it is considered to be the most difficult paradoxical complex. It is well known that biobased polymer materials have significant gaps in mechanical strength compared to metallic materials. In order to ensure the long-term efficacy of biodegradable vascular scaffolds *in vivo*, it is necessary to increase their thickness accordingly.^99^ However, the increase in thickness extensively increases the probability of late revascularization.^100^ Therefore, the conflict between the acquisition of mechanical properties of biodegradable vascular scaffolds and the subsequent occurrence of restenosis has become an urgent medical problem. To overcome this problem and achieve the safety and efficiency of biodegradable vascular scaffolds, the only solution is to find appropriate strategies to resolve or eliminate this conflict as soon as possible.

### 2.4. Macromolecular drugs and drug controlled release polymer materials

A class of medical functional polymer materials with pharmacological effects that react with human physiological tissues and lose their medicinal properties after degradation to small molecules is known as macromolecular drugs. Common synthetic polymer drugs include polystyrene trimethylbenzyl ammonium (antibacterial drugs), maleic anhydride copolymers (antiviral drugs) and polyamino acid polymers (anticancer drugs). In order to improve the drug efficiency, reduce toxicity and side effects, alleviate patient suffering, and shorten administration time, biodegradable drug controlled release polymer materials have been developed extensively. The so-called controlled release of drugs simply involves utilizing the dissolution, diffusion, permeation, and ion exchange characteristics of drugs, employing appropriate excipients or carrier tools (such as polymer matrices, capsule shells, diluents, lubricants, and binders). Tumor cells exist in a unique microenvironment that typically has a relatively high temperature, a specific enzyme system and a relatively low pH.^101–104^ Drug controlled release systems can precisely take advantage of the differences in microenvironment between tumor tissue and normal tissue to intelligently release drugs to tumor cell targets.^105^

**Fig. 10:**
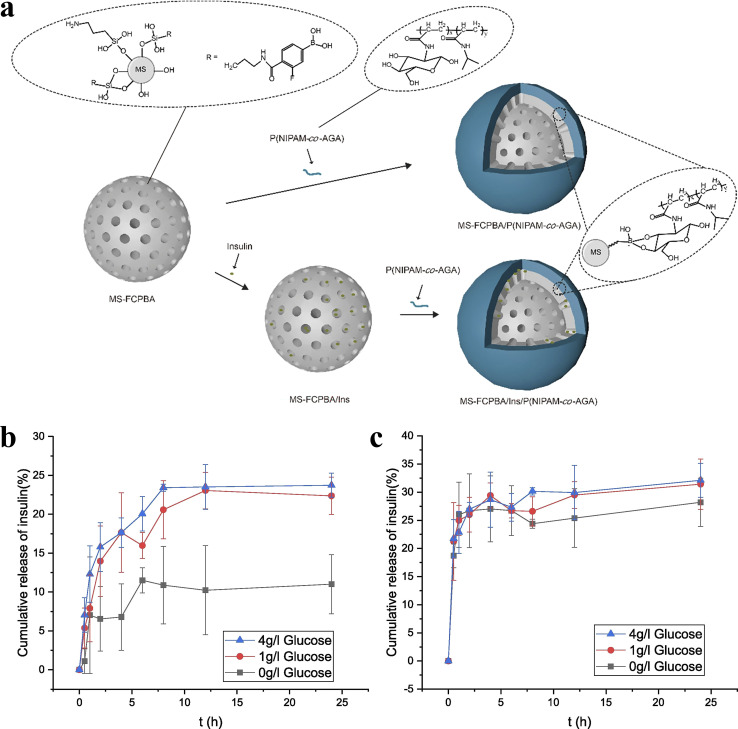
(a) Schematic illustration of the preparation of MS-FCPBA/P(NIPAM-*co*-AGA) and MS-FCPBA/Ins/P(NIPAM-*co*-AGA). (b) Cumulative release of insulin from MS-FCPBA/P(NIPAM-*co*-AGA) in different glucose concentrations at 37 °C. (c) Cumulative release of insulin from MS-FCPBA/P(AAc-*co*-AGA) in different glucose concentrations at 37 °C. Reproduced from ref. 106 with permission from Elsevier, copyright 2021.

Huang *et al.*^106^ modified mesoporous silica (MS) with 3-fluoro-4-carboxyphenylboronic acid (FCPBA) to obtain mesoporous silica modified with phenylboronic acid (MS-FCPBA). *N*-Acryloylglucosamine (AGA) was copolymerized with *N*-isopropylacrylamide (NIPAM) and acrylic acid (AAc), respectively, to synthesize two polymers with diol groups. They are noted as P(NIPAM-*co*-AGA) and P(AAc-*co*-AGA) in turn. Due to the formation of borate ester bonds between the phenylboronic acid group and the diol group, P(NIPAM-*co*-AGA) and P(AAc-*co*-AGA) were coated on the surface of MS-FCPBA containing insulin, resulting in the insulin release system. Fig. 10 illustrates the preparation schemes for MS-FCPBA/P (NIPAM-*co*-AGA) and MS-FCPBA/Ins/P (NIPAM-*co*-AGA), while the schemes for MS-FCPBA/P(AAc-*co*-AGA) and MS-FCPBA/Ins/P(AAc-*co*-AGA) are nearly identical. The MS-FCPBA/P (NIPAM-*co*-AGA) samples showed more pronounced glucose responsiveness at three glucose concentrations (0, 1, and 4 g L^−1^) compared to the MS-FCPBA/P (AAc-*co*-AGA) samples, aimed at investigating the cumulative release of glucose-responsive insulin, where insulin release was significantly higher in the presence of glucose than without (though some insulin was released even without glucose). The reason for the difference in glucose-responsiveness is that P(AAc-*co*-AGA) is a relatively hydrophilic polymer that is easily shed in an aqueous environment, ultimately leading to drug release. In contrast, P(NIPAM-*co*-AGA) is a polymer containing hydrophobic groups, and there may be a combination of hydrophobic waters between different P(NIPAM-*co*-AGA) molecules, which makes P(NIPAM-*co*-AGA) tend to adhere tightly to the surface of MS-FCPBA. As a result, desorption is more difficult to occur and some drugs can be well preserved in a glucose-free environment.

Ebrahimifar *et al.*^107^ used a mixed matrix of PCL, PLA and polyvinyl cyclohexane carbonate (PVCHC) as carriers for the hydrophilic drugs acetaminophen and clindamycin to explore their effect on drug release rate. The authors observed that in all drug-polymer matrices, acetaminophen and clindamycin were released in large amounts from the first hour, which may be due to the physical adsorption of drug molecules on the surface of polymer microparticles. Furthermore, the higher drug concentration released from clindamycin-loaded matrices compared to acetaminophen-loaded matrices was due to the physical adsorption of clindamycin particles onto the surface of the matrices *via* van der Waals forces. However, the acetaminophen polymeric matrix showed higher release efficiency compared to the acetaminophen polymeric matrix. A plausible explanation is the weak interaction between the amine and hydroxyl groups of acetaminophen and the ester and carbonyl groups in the polymer. Furthermore, it was found that PVCHC with shorter chain length than PCL and PLA could affect the interception of acetaminophen and clindamycin molecules and increase the barrier of drug molecules between the polymer layers, resulting in similar drug release efficiency of acetaminophen and clindamycin on PLA-PVCHC.

Su *et al.*^108^ grafted PH-sensitive amphiphilic poly(2-(diethylamino)ethyl methacrylate (PDEAEMA)) on the surface of mesoporous silica molecular sieve SBA-15 (SBA-15-*g*-PDEAEMA) and then loaded the anticancer drug quercetin (Qu) on it (SBA-15-*g*-PDEAEMA-Qu) to study the controlled Qu release. The weakly basic tertiary amine groups contained in the side chains of PDEAEMA can be protonated in an acidic environment, thus allowing the molecular chains of PDEAEMA to be elongated in acidic aqueous solutions due to the mutual repulsion of charges. However, in a neutral or alkaline environment, the protonation is weakened or even disappears, producing a phenomenon in which the attraction between molecular chains is greater than the repulsion between charges, which causes the polymer chains to curl and contract. Thus, SBA-15-*g*-PDEAEMA is well dispersed in acidic conditions, slightly dispersed in neutral conditions, and aggregated in alkaline conditions, indicating a good pH sensitivity of the material. Because of this, Qu is released slowly under normal pH conditions of human tissues (pH = 7.4), with a maximum release rate of only 38%, while under pH conditions of tumor cells (pH = 5.5), the release rate is much faster, reaching a maximum of 77% at 96 h. Therefore, SBA-15-*g*-PDEAEMA has potential applications in clinical drug release control.

During drug delivery, researchers always pay attention to the drug loading capacity, drug release rate and targeting, however, these performance parameters are entirely achieved by the drug-controlled release polymer material itself.^109^ Numerous experiments have demonstrated that many drug-controlled release polymer materials have made great progress in these aspects through modification techniques, but in fact, there are not many ideal drug-controlled release polymer materials that can integrate these properties at the same time. More importantly, there are still problems in non-core aspects that deserve our in-depth study. For example, the effect of bulk polymer degradation on drug release is often overlooked and reports on it are rarely heard. For better and faster development of drug-controlled release polymeric materials, we should focus on the core issues and their opposites and keep them in sync.

## 3. Challenges

In recent years, there are many researchers working to break through the limitations of BMPMs in clinical practice, and great progress has been made. However, there are still some studies that claim most BMPMs have some drawbacks, such as poor biocompatibility, uncontrollable degradation rate, few varieties. In other words, BMPMs are facing many serious challenges, this greatly hinders the day when they can effectively serve humanity.

### 3.1. The material itself

#### 3.1.1. Poor biocompatibility

For the organism itself, the implanted BMPMs are always foreign substances. Due to instinctive self-protection, the organism reacts with rejection, and the severity of the reaction characterizes the biocompatibility of BMPMs. Depending on the different uses of BMPMs in living organisms, biocompatibility can be divided into two types: histocompatibility and hemocompatibility. For the former, some polymer materials themselves (polyurethane, polyvinyl chloride, *etc.*) have no adverse effects on diseased tissues, but during the actual processing and synthesis, small amounts of impurities, or some additives, residual monomers and oligomers, are inevitably mixed in. When stents made of these materials are implanted in the diseased body, these impurities slowly migrate from the interior to the surface, thus reacting with the surrounding tissue and causing inflammation or tissue deformation, or even systemic reactions. In view of the latter, the antithrombotic problem has been a pressing clinical issue for BMPMs. Although many articles have proposed and demonstrated that grafting compounds with anticoagulant properties (heparin, polyoxyethylene, *etc.*) onto the material surface by coupling or plasma methods can effectively inhibit the formation of thrombus around the material, this problem has not been completely solved, as the results of subsequent studies have shown that small areas of thrombus are still present on the surface of the modified material.

#### 3.1.2. Uncontrollable rate of material degradation

How to accurately control the degradation rate of BMPMs is also a challenge in clinical applications. Theoretically, the degradation rate of BMPMs should be equal to the rate of tissue regeneration, which can be considered as an optimal match. Of course, it allows for a slight gap. And once this slight red line is breached, it can lead to the failure of BMPMs implantation. If the degradation rate of BMPMs is much higher than the rate of tissue regeneration, it is not conducive to the adhesion of new tissues on the surface of the material, and the osseointegration cannot be well formed in the area of BMPMs implantation, making it difficult to induce osteogenesis, which will eventually lead to local collapse or even reconstruction failure. On the contrary, the stress shielding caused by delayed degradation will affect the growth of new tissues. Therefore, we know that it is urgent to solve the matching problem in order to make BMPMs accepted from theory to reality as soon as possible.

#### 3.1.3. Single-species materials

Statistically, very few BMPMs have been certified to exert significant medical effects so far. At present, the materials utilized by researchers in clinical trials are mostly PLA-based, PC-based, PGA-based and PCL-based, and rarely involve the development of new materials, which leads to the lack of varieties of BMPMs. Meanwhile, the existing varieties are relatively homogeneous, which is not conducive to the horizontal development of BMPMs. The lack of new materials is the main factor that makes it difficult for BMPMs to take root in the clinic, so we must increase the efforts to explore new materials and improve the speed in time.

### 3.2. Lack of accurate and effective techniques

Technological methods also play a crucial role in the development of BMPMs. Only with the help of technological platforms can raw materials show their excellent properties while achieving complex shape designs. 3D printing technology is one of the most commonly used auxiliary methods for preparing medical products and is well established in domestic industrial manufacturing, which allows products using 3D printing technology to have a high degree of precision. However, this precision has not been fully accepted by medicine. The specific reason is that the current 3D printing technology can only process simple tissues in medicine, and it is difficult to print organs or tissues with complex structures and physiological functions. More importantly, organs with complex structures also lengthen the printing time significantly. Therefore, we can conclude that the lack of high-precision and high-efficiency technology also limits the development of BMPMs to a certain extent.

## 4. Solutions and prospects

In view of the many factors that seriously hinder the development of BMPMs mentioned above, we propose some reasonable solutions and scientifically look forward to their future development, which will promote the better and faster development of BMPMs.

### 4.1. Solutions

#### 4.1.1. Clarify the current problems

To break the pattern of disconnection between BMPM theory and practical application, the first step is to develop the material itself, including two aspects. On the one hand, on the premise of ensuring that the original advantages do not decline, the existing materials should be enhanced and modified to achieve optimization for their shortcomings. For example, improvements can be made by immobilizing heparin on the surface of materials with poor antithrombotic properties. Another is to vigorously search for new materials that address clinical needs, and then work to enrich the range of materials. Second, the improvement of technical methods can accelerate the development of BMPMs. What we should do is to actively guide 3D printing technology to improve in the direction of high precision and efficiency in order to achieve better integration with BMPMs in the medical field as early as possible.

#### 4.1.2. Tapping the existing blind spots

Although the research process of BMPMs has many participants and lasts for a long time, there are still some blind spots that have not been discovered and paid attention to. Exploration of the blind spots can enable researchers to fully grasp the performance changes of BMPM and thus fill the gaps in development, which also provides some guiding suggestions for researchers.

##### 4.1.2.1. Dynamic mechanical behavior during material degradation

Researchers have always paid great attention to the static mechanical strength of BMPMs before implantation, and they are still working on this issue. However, it is worth emphasizing that the dynamic changes of mechanical properties during material degradation are of higher importance, which cannot be ignored because we can observe the relationship between the mechanical strength of the material and its degradation time or degradation rate. On this basis, we can purposefully synthesize a new material whose mechanical properties do not weaken significantly during the degradation phase, providing a longer and stronger support for the disease body. Only by combining static and dynamic mechanical behaviors can we more accurately promote the development of BMPMs.

##### 4.1.2.2. Safety of by-products

Since the preparation of BMPMs is difficult to achieve high purity, small amounts of impurity substances such as monomers and oligomers as well as degradation products present in BMPMs are bound to be released after a period of implantation into the patient. What kind of effect these substances have on the organism and whether the degree of effect is serious (*e.g.* whether they are toxic or not, whether they can induce cancer, *etc.*), there is no accurate conclusion in medical science and further confirmation is needed.

### 4.2. Future directions

#### 4.2.1. Materials with self-growth properties

At present, most experimental subjects of bone repair and regeneration research are mainly middle-aged and elderly patients, while there are few reports on adolescents. Adolescents also need the help of artificial bone due to massive bone defects caused by accidents or major diseases. The biggest difference is that for middle-aged and elderly patients whose bones are largely set, adolescents have a faster bone growth rate during the developmental phase, which makes it necessary for the material to meet the existing requirements with a certain degree of self-growth. Unfortunately, BMPMs do not contain the growth factors required for human growth. We can replace them by stretching or migration of molecular chains in the polymer to achieve artificial bone growth. The triggering methods can be designed as light irradiation, magnetic attraction and thermal radiation. The development of self-growing materials is of great value and significance for the treatment of adolescent patients.

#### 4.2.2. Smart materials

Smart will definitely evolve into the mainstream trend of BMPMs in the future. Imagine an environment where one can intelligently manipulate the degradation rate of BMPMs with the aid of light, temperature and UV light. The higher the intensity, the faster the degradation rate of BMPMs. How convenient it is to deal with the matching problem between material degradation and tissue formation. Likewise, the problem of drug delivery carriers for effective targeted therapies can be easily solved. As a result, smart BMPMs are bound to provide diverse therapeutic avenues for patients in the clinic through innovative construction, material enhancement and customized drug delivery.^110^

## 5. Conclusion

In summary, BMPMs play a considerable role in the medical field with their unique advantages, and in recent years, they have made great progress with advanced manufacturing technology, which has greatly promoted the development of BMPMs.

Although BMPMs show great potential in biomedicine, they have some inherent disadvantages. For natural BMPMs, they are difficult to access since they are found in plants and animals. Secondly, the mechanical properties of these materials are low, and their products usually fail to withstand large loads during service. For synthetic BMPMs, they are available, but the surface activity of such materials is poor and they often do not actively in the incorporation with native tissues. In addition, some synthetic BMPMs, especially PLA and PCL, tend to exhibit strong hydrophobicity (with contact angles much greater than 90°), which is not conducive to cellular adherence and multiplication. Therefore, in order for BMPMs to flourish in clinical practice, the only thing we have to do now is to do our best to overcome the complex challenges we are facing.

## Data availability

No primary research results, software or code have been included and no new data were generated or analysed as part of this review.

## Author contributions

Fulong Li was responsible for investigation, writing/review, and editing. Chao Chen contributed to conceptualization, resources, writing/original draft, and revision.

## Conflicts of interest

The authors declare that they have no known competing financial interests or personal relationships that could have appeared to influence the work reported in this paper.

## References

[cit1] Thalji M. R., Ibrahim A. A., Ali G. A. M. (2021). Cutting-edge development in dendritic polymeric materials for biomedical and energy applications. Eur. Polym. J..

[cit2] Lu Z., Wu Y., Cong Z., Qian Y., Wu X., Shao N., Qiao Z., Zhang H., She Y., Chen K., Xiang H., Sun B., Yu Q., Yuan Y., Lin H., Zhu M., Liu R. (2021). Effective and biocompatible antibacterial surfaces via facile synthesis and surface modification of peptide polymers. Bioact. Mater..

[cit3] Yang X., Li Y., He W., Huang Q., Zhang R., Feng Q. (2018). Hydroxyapatite/collagen coating on PLGA electrospun fibers for osteogenic differentiation of bone marrow mesenchymal stem cells. J. Biomed. Mater. Res., Part A.

[cit4] George S. M., Kandasubramanian B. (2020). Advancements in MXene-Polymer composites for various biomedical applications. Ceram. Int..

[cit5] Palodkar K. K., Rao N. N. M., Iyer S., Puttalingaiah R. T., Sadhu V., Aminabhavi T. M., Reddy K. R., Sainath A. V. S. (2022). Maltose-based methacrylated polymer architectures and their biocompatibility. Mater. Today Chem..

[cit6] Kalva N., Uthaman S., Lee S. J., Lim Y. J., Augustine R., Huh K. M., Park I. K., Kim I. (2021). Degradable pH-responsive polymer prodrug micelles with aggregation-induced emission for cellular imaging and cancer therapy. React. Funct. Polym..

[cit7] Demina V. A., Krasheninnikov S. V., Buzin A. I., Kamyshinsky R. A., Sadovskaya N. V., Goncharov E. N., Zhukova N. A., Khvostov M. V., Pavlova A. V., Tolstikova T. G., Sedush N. G., Chvalun S. N. (2020). Biodegradable poly(L-lactide)/calcium phosphate composites with improved properties for orthopedics: Effect of filler and polymer crystallinity. Mater. Sci. Eng., C.

[cit8] Lett J. A., Sagadevan S., Fatimah I., Hoque M. E., Lokanathan Y., Leonard E., Alshahateet S. F., Schirhagl R., Oh W. C. (2021). Recent advances in natural polymer-based hydroxyapatite scaffolds: Properties and applications. Eur. Polym. J..

[cit9] Wu T., Liu W., Huang S., Chen J., He F., Wang H., Zheng X., Li Z., Zhang H., Zha Z., Lin Z., Chen Y. (2021). Bioactive strontium ions/ginsenoside Rg1-incorporated biodegradable silk fibroin-gelatin scaffold promoted challenging osteoporotic bone regeneration. Mater. Today Bio.

[cit10] Beheshtizadeh N., Lotfibakhshaiesh N., Pazhouhnia Z., Hoseinpour M., Nafari M. (2020). A review of 3D bio-printing for bone and skin tissue engineering: a commercial approach. J. Mater. Sci..

[cit11] Matai I., Kaur G., Seyedsalehi A., McClinton A., Laurencin C. T. (2020). Progress in 3D bioprinting technology for tissue/organ regenerative engineering. Biomaterials.

[cit12] Rangel A., Colaco L., Nguyen N. T., Grosset J. F., Egles C., Migonney V. (2022). Adapting mechanical characterization of a biodegradable polymer to physiological approach of anterior cruciate ligament functions. IRBM.

[cit13] Du Z., Leng H., Guo L., Huang Y., Zheng T., Zhao Z., Liu X., Zhang X., Cai Q., Yang X. (2020). Calcium silicate scaffolds promoting bone regeneration via the doping of Mg^2+^ or Mn^2+^ ion. Composites, Part B.

[cit14] Farzin A., Hassan S., Ebrahimi-Barough S., Ai A., Hasanzadeh E., Goodarzi A., Ai J. (2019). A facile two step heat treatment strategy for development of bioceramic scaffolds for hard tissue engineering applications. Mater. Sci. Eng., C.

[cit15] Torres T. G., Nazhat S. N., Fadzullah S. H. S. M., Maquet V., Boccaccini A. R. (2007). Mechanical properties and bioactivity of porous PLGA/TiO2 nanoparticle-filled composites for tissue engineering scaffolds. Compos. Sci. Technol..

[cit16] Bouet D., Marchat M., Cruel L., Malaval L., Vico L. (2015). In vitro three-dimensional bone tissue models: From cells to controlled and dynamic environment. Tissue Eng., Part B.

[cit17] Xu C., Liu Z., Chen X., Gao Y., Wang W., Zhuang X., Zhang H., Dong X. (2024). Bone tissue engineering scaffold materials: Fundamentals, advances, and challenges. Chin. Chem. Lett..

[cit18] Sobczak-Kupiec A., Olender E., Malina D., Tyliszczak B. (2018). Effect of calcination parameters on behavior of bone hydroxyapatite in artificial saliva and its biosafety. Mater. Chem. Phys..

[cit19] Lin M., Sun J. (2022). Antimicrobial peptide-inspired antibacterial polymeric materials for biosafety. Biosafety Health.

[cit20] Wu H., Zhang R., Hu B., He Y., Zhang Y., Cai L., Wang L., Wang G., Hou H., Qiu X. (2021). A porous hydrogel scaffold mimicking the extracellular matrix with swim bladder derived collagen for renal tissue regeneration. Chin. Chem. Lett..

[cit21] Armentano I., Dottori M., Fortunati E., Mattioli S., Kenny J. M. (2010). Biodegradable polymer matrix nanocomposites for tissue engineering: A review. Polym. Degrad. Stab..

[cit22] Webber M. J., Khan O. F., Sydlik S. A., Tang B. C., Langer R. (2015). A perspective on the clinical translation of scaffolds for tissue engineering. Ann. Biomed. Eng..

[cit23] Hutmacher D. W. (2000). Scaffolds in tissue engineering bone and cartilage. Biomaterials.

[cit24] Turnbull G., Clarke J., Picard F., Riches P., Jia L., Han F., Li B., Shu W. (2018). 3D bioactive composite scaffolds for bone tissue engineering. Bioact. Mater..

[cit25] Cui J., Ren L., Mai J., Zheng P., Zhang L. (2022). 3D Printing in the context of cloud manufacturing. Robot. Comput. Integrated Manuf..

[cit26] Liu C., Xu N., Zong Q., Yu J., Zhang P. (2021). Hydrogel prepared by 3D printing technology and its applications in the medical field. Colloid Interface Sci. Commun..

[cit27] Yan Q., Dong H., Su J., Han J., Song B., Wei Q., Shi Y. (2018). A review of 3D printing technology for medical applications. Engineering.

[cit28] Liu J., Sun L., Xu W., Wang Q., Yu S., Sun J. (2019). Current advances and future perspectives of 3D printing natural-derived biopolymers. Carbohydr. Polym..

[cit29] Lindquist E. M., Gosnell J. M., Khan S. K., Byl J. L., Zhou W., Jiang J., Vettukattil J. J. (2021). 3D printing in cardiology: A review of applications and roles for advanced cardiac imaging. Ann. 3D Print. Med..

[cit30] Chong W. J., Shen S., Li Y., Trinchi A., Pejak D., Kyratzis I., Sola A., Wen C. (2022). Additive manufacturing of antibacterial PLA-ZnO nanocomposites: Benefits, limitations and open challenges. J. Mater. Sci. Technol..

[cit31] Puppi D., Chiellini F. (2020). Biodegradable polymers for biomedical additive manufacturing. Appl. Mater. Today.

[cit32] Singh N., Singh G. (2021). Advances in polymers for bio-additive manufacturing: A state of art review. J. Manuf. Process..

[cit33] Khalaj R., Tabriz A. G., Okereke M. I., Douroumis D. (2021). 3D printing advances in the development of stents. Int. J. Pharm..

[cit34] Shanmugam V., Das O., Babu K., Marimuthu U., Veerasimman A., Johnson D. J., Neisiany R. E., Hedenqvist M. S., Ramakrishna S., Berto F. (2021). Fatigue behaviour of FDM-3D printed polymers, polymeric composites and architected cellular materials. Int. J. Fatig..

[cit35] Melnyk L. A., Oyewumi M. O. (2021). Integration of 3D printing technology in pharmaceutical compounding: Progress, prospects, and challenges. Ann. 3D Print. Med..

[cit36] Jandyal A., Chaturvedi I., Wazir I., Raina A., Ul Haq M. I. (2022). 3D printing - A review of processes, materials and applications in industry 4.0. Sustain. Oper. Comput..

[cit37] Gao C., Lu C., Jian Z., Zhang T., Chen Z., Zhu Q., Tai Z., Liu Y. (2021). 3D bioprinting for fabricating artificial skin tissue. Colloids Surf., B.

[cit38] Sun S., Fei G., Wang X., Xie M., Guo Q., Fu D., Wang Z., Wang H., Luo G., Xia H. (2021). Covalent adaptable networks of polydimethylsiloxane elastomer for selective laser sintering 3D printing. Chem. Eng. J..

[cit39] Zhu J., Wu P., Chao Y., Yu J., Zhu W., Liu Z., Xu C. (2022). Recent advances in 3D printing for catalytic applications. Chem. Eng. J..

[cit40] Teoh J. H., Tay S. M., Fuh J., Wang C. H. (2022). Fabricating scalable, personalized wound dressings with customizable drug loadings via 3D printing. J. Controlled Release.

[cit41] Haider S. H., Kummara M. R., Kamal T., Alghyamah A. A. A., Iftikhar F. J., Bano B., Khan N., Afridi M. A., Han S. S., Alrahlah A., Khan R. (2020). Advances in the scaffolds fabrication techniques using biocompatible polymers and their biomedical application: A technical and statistical review. J. Saudi Chem. Soc..

[cit42] Generalova N., Vikhrov A. A., Prostyakova A. I., Apresyan S., Stepanov A. G., Myasoedov M. S., Oleinikov V. A. (2024). Polymers in 3D printing of external maxillofacial prostheses and in their retention systems. Int. J. Pharm..

[cit43] Arora N., Dua S., Singh V. K., Singh S. K., Senthilkumar T. (2024). A comprehensive review on fillers and mechanical properties of 3D printed polymer composites. Mater. Today Commun..

[cit44] Bellia-Munzon G., Cieri P., Toselli L., Cuestas G., Doormann F., Gabaldon-Masse P., Rodriguez V., Bellia-Munzon P. (2021). Resorbable airway splint, stents, and 3D reconstruction and printing of the airway in tracheobronchomalacia. Semin. Pediatr. Surg..

[cit45] Okereke M. I., Khalaj R., Tabriz A. G., Douroumis D. (2021). Development of 3D printable bioresorbable coronary artery stents: A virtual testing approach. Mech. Mater..

[cit46] Baniasadi H., Madani Z., Ajdary R., Rojas O. J., Seppala J. (2021). Ascorbic acid-loaded polyvinyl alcohol/cellulose nanofibril hydrogels as precursors for 3D printed materials. Mater. Sci. Eng., C.

[cit47] Lin M., Lin J., Huang C., Chen Y. (2020). Textile fabricated biodegradable composite stents with core-shell structure. Polym. Test..

[cit48] Herath S. S., Downing D., Cometta S., Tino R., Castro N. J., Leary M., Schmutz B., Wille M. L., Hutmacher D. W. (2021). Mechanical and geometrical study of 3D printed Voronoi scaffold design for large bone defects. Mater. Des..

[cit49] Tang P., Song P., Peng Z., Zhang B., Gui X., Wang Y., Liao X., Chen Z., Zhang Z., Fan Y., Li Z., Cen Y., Zhou C. (2021). Chondrocyte-laden GelMA hydrogel combined with 3D printed PLA scaffolds for auricle regeneration. Mater. Sci. Eng., C.

[cit50] Botvin V., Karaseva S., Salikova D., Dusselier M. (2021). Syntheses and chemical transformations of glycolide and lactide as monomers for biodegradable polymers. Polym. Degrad. Stab..

[cit51] Ansari I., Singh P., Mittal A., Mahato R. I., Chitkara D. (2021). 2,2-Bis(hydroxymethyl) propionic acid based cyclic carbonate monomers and their (co)polymers as advanced materials for biomedical applications. Biomaterials.

[cit52] Hayat U., Ali R., Bilal M., Iqbal H. M. N., Wang J. (2022). Biodegradable polymeric conduits: Platform materials for guided nerve regeneration and vascular tissue engineering. J. Drug Deliv. Sci. Technol..

[cit53] Jan N., Madni A., Rahim M. A., Khan N. U., Jamshaid T., Khan A., Jabar A., Khan S., Shah H. (2021). In vitro anti-leukemic assessment and sustained release behaviour of cytarabine loaded biodegradable polymer based nanoparticles. Life Sci..

[cit54] Mansouri N., Al-Sarawi S., Losic D., Mazumdar J., Clark J., Gronthos S., Doig R. O. (2021). Biodegradable and biocompatible graphene-based scaffolds for functional neural tissue engineering: A strategy approach using dental pulp stem cells and biomaterials. Biotechnol. Bioeng..

[cit55] Bachtiar E. O., Ritter V. C., Gall K. (2021). Structure-property relationships in 3D-printed poly(l-lactide-co-ε-caprolactone) degradable polymer. J. Mech. Behav. Biomed. Mater..

[cit56] Thadepalli S. (2022). Review of multifarious applications of polymers in medical and health care textiles. Mater. Today: Proc..

[cit57] Ragunathan S., Govindasamy G., Raghul D. R., Karuppaswamy M., VijayachandraTogo R. K. (2020). Hydroxyapatite reinforced natural polymer scaffold for bone tissue regeneration. Mater. Today: Proc..

[cit58] Wang S., Huang Z., Liu L., Shi Z., Liu J., Li Z., Hao Y. (2021). Design and study of in vivo bone formation characteristics of biodegradable bioceramic. Mater. Des..

[cit59] Li F., Chen X., Liu P. (2023). A review on three-dimensional printed silicate-based bioactive glass/biodegradable medical synthetic polymer composite scaffolds. Tissue Eng., Part B.

[cit60] Moetazedian A., Gleadall A., Mele E., Silberschmidt V. V. (2021). Damage in extrusion additive manufactured biomedical polymer: Effects of testing direction and environment during cyclic loading. J. Mech. Behav. Biomed. Mater..

[cit61] Molnar L. M., Delgado Sobrino D. R., Lecky S., Michal D. (2019). Medical applications of biomaterials: The case of design and manufacture of orthopedic corsets made of polylactic acid by additive manufacturing. Mater. Sci. Forum.

[cit62] Ebrahimi F., Dana H. R. (2022). Poly lactic acid (PLA) polymers: from properties to biomedical applications. Int. J. Polym. Mater..

[cit63] Silva D., Kaduri M., Poley M., Adir O., Krinsky N., Shainsky-Roitman J., Schroeder A. (2018). Biocompatibility, biodegradation and excretion of polylactic acid (PLA) in medical implants and theranostic systems. Chem. Eng. J..

[cit64] Tumer H., Erbil H. Y. (2021). Extrusion-based 3D printing applications of PLA composites: A review. Coatings.

[cit65] Wang W., Ye G., Fan D., Lu Y., Shi P., Wang X., Bateer B. (2021). Photo-oxidative resistance and adjustable degradation of poly-lactic acid (PLA) obtained by biomass addition and interfacial construction. Polym. Degrad. Stab..

[cit66] Huang Y., Bruenig H., Boldt R., Mueller M. T., Wiessner S. (2022). Fabrication of melt-spun fibers from irradiation modified biocompatible PLA/PCL blends. Eur. Polym. J..

[cit67] Senthamaraikannan K. A., Amanullah S., Barath M., Manojkumar R., Jagadeeshwaran J. (2020). Overview of Polylactic acid and its derivatives in medicinal applications. Mater. Sci. Eng..

[cit68] Polak-Krasna K., Abaei A. R., Shirazi R. N., Parle E., Carroll O., Ronan W., Vaughan T. J. (2021). Physical and mechanical degradation behaviour of semi-crystalline PLLA for bioresorbable stent applications. J. Mech. Behav. Biomed. Mater..

[cit69] Chen X., Chen G., Wang G., Zhu P., Gao C. (2020). Recent progress on 3D-printed polylactic acid and its applications in bone repair. Adv. Eng. Mater..

[cit70] Farah S., Anderson D. G., Langer R. (2016). Physical and mechanical properties of PLA, and their functions in widespread applications - A comprehensive review. Adv. Drug Delivery Rev..

[cit71] Elsawy M. A., Kim K. H., Park J. W., Deep A. (2017). Hydrolytic degradation of polylactic acid (PLA) and its composites. Renewable Sustainable Energy Rev..

[cit72] Chihaoui Q. T., Delgado-Aguilar M., Mutje P., Boufi S. (2022). Lignin-containing cellulose fibrils as reinforcement of plasticized PLA biocomposites produced by melt processing using PEG as a carrier. Ind. Crops Prod..

[cit73] Acik G. (2020). Preparation of antimicrobial and biodegradable hybrid soybean oil and poly (L-lactide) based polymer with quaternized ammonium salt. Polym. Degrad. Stab..

[cit74] Gremare A., Guduric V., Bareille R., Heroguez V., Latour S., L'Heureux N., Fricain J. C., Catros S., Le Nihouannen D. (2018). Characterization of printed PLA scaffolds for bone tissue engineering. J. Biomed. Mater. Res., Part A.

[cit75] Kushwanth Theja K., Bharathiraja G., Sakthi Murugan V., Muniappan A. (2023). Evaluation of mechanical properties of tea dust filler reinforced polymer composite. Mater. Today: Proc..

[cit76] Eghbalian M., Ansari R., Haghighi S. (2022). On the mechanical properties and fracture analysis of polymer nanocomposites reinforced by functionalized silicon carbide nanotubes: A molecular dynamics investigation. J. Mol. Graphics Modell..

[cit77] Bano N., Jikan S. S., Basri H., Adzila S., Badarulzaman N. A. (2019). Physicomechanical properties of nanobiocomposite composed of polylactic acid and biogenic nano hydroxyapatite. Int. J. Integr. Eng..

[cit78] Wan M., Liu S., Huang D., Qu Y., Hu Y., Su Q., Zheng W., Dong X., Zhang H., Wei Y., Zhou W. (2021). Biocompatible heterogeneous bone incorporated with polymeric biocomposites for human bone repair by 3D printing technology. J. Appl. Polym. Sci..

[cit79] Wang W., Zhang B., Li M., Li J., Zhang C., Han Y., Wang L., Wang K., Zhou C., Liu L., Fan Y., Zhang X. (2021). 3D printing of PLA/n-HA composite scaffolds with customized mechanical properties and biological functions for bone tissue engineering. Composites, Part B.

[cit80] Rizvi S. H. A., Jiale C., Mehboob A., Zaheer U., Chang S. H. (2021). Experimental study on magnesium wire-polylactic acid biodegradable composite implants under in vitro material degradation and fatigue loading conditions. Compos. Struct..

[cit81] Mehboob A., Mehboob H., Chang S. H. (2020). Evaluation of unidirectional BGF/PLA and Mg/PLA biodegradable composites bone plates-scaffolds assembly for critical segmental fractures healing. Composites, Part A.

[cit82] Ali W., Mehboob A., Han M., Chang S. H. (2019). Effect of fluoride coating on degradation behaviour of unidirectional Mg/PLA biodegradable composite for load-bearing bone implant application. Composites, Part A.

[cit83] Jiang D. and Ning F., Fused filament fabrication of biodegradable PLA/316L composite scaffolds: Effects of metal particle content, 48th SME North American Manufacturing Research Conference, 2000, 48, pp. 755–762

[cit84] Chen T., Zou Q., Du C., Wang C., Li Y., Fu B. (2020). Biodegradable 3D printed HA/CMCS/PDA scaffold for repairing lacunar bone defect. Mater. Sci. Eng., C.

[cit85] Rodriguez-Zapater S., Serrano-Casorran C., Guirola J. A., Lopez-Minguez S., Bonastre C., de Gregorio M. A. (2020). Reactivity study of a biodegradable polydioxanone tracheal stent in a rabbit model. Arch. Bronconeumol..

[cit86] Zhang Y., Liu Y., Jiang Z., Wang J., Xu Z., Meng K., Zhao H. (2021). Poly(glyceryl sebacate)/silk fibroin small-diameter artificial blood vessels with good elasticity and compliance. Smart Mater. Med..

[cit87] Zhou L., Zheng H., Wang S., Zhou F., Lei B., Zhang Q. (2020). Biodegradable conductive multifunctional branched poly(glycerol-amino acid)-based scaffolds for tumor/infection-impaired skin multimodal therapy. Biomaterials.

[cit88] Lo C. H., Brown J. N., Dantzer E. J. G., Maitz P. K. M., Vandervord J. G., Wagstaff M. J. D., Barker T. M., Cleland H. (2022). Wound healing and dermal regeneration in severe burn patients treated with NovoSorb^®^ Biodegradable Temporising Matrix: A prospective clinical study. Burns.

[cit89] Morsada Z., Hossain M. M., Islam M. T., Mobin M. A., Saha S. (2021). Recent progress in biodegradable and bioresorbable materials: From passive implants to active electronics. Appl. Mater. Today.

[cit90] Johnbosco C., Zschoche S., Nitschke M., Hahn D., Werner C., Maitz M. F. (2021). Bioresponsive starPEG-heparin hydrogel coatings on vascular stents for enhanced hemocompatibility. Mater. Sci. Eng., C.

[cit91] Anderloni A., Fugazza A., Maroni L., Ormando V., Maselli R., Carrara S., Cappello A., Mangiavillano B., Omodei P., Preatoni P., Galtieri P. A., Pellegatta G., Repici A. (2020). New biliary and pancreatic biodegradable stent placement: a single-center, prospective, pilot study. Gastrointest. Endosc..

[cit92] Yin T., Du R., Wang Y., Huang J., Ge S., Huang Y., Tan Y., Liu Q., Chen Z., Feng H., Du J., Wang Y., Wang G. (2022). Two-stage degradation and novel functional endothelium characteristics of a 3-D printed bioresorbable scaffold. Bioact. Mater..

[cit93] Sane M., Dighe V., Patil R., Hassan P. A., Gawali S., Patravale V. (2021). Bivalirudin and sirolimus co-eluting coronary stent: Potential strategy for the prevention of stent thrombosis and restenosis. Int. J. Pharm..

[cit94] Toh H. W., Toong D. W. Y., Ng J. C. K., Ow V., Lu S., Tan L. P., Wong P. E. H., Venkatraman S., Huang Y., Ang H. (2021). Polymer blends and polymer composites for cardiovascular implants. Eur. Polym. J..

[cit95] Hytonen J. P., Taavitsainen J., Tarvainen S., Yla-Herttuala S. (2018). Biodegradable coronary scaffolds: their future and clinical and technological challenges. Cardiovasc. Res..

[cit96] Khalaj Amnieh S., Mashayekhi M., Shahnooshi E., Tavafoghi M., Mosaddegh P. (2021). Biodegradable performance of PLA stents affected by geometrical parameters: The risk of fracture and fragment separation. J. Biomech..

[cit97] Lee S. J., Jo H. H., Lim K. S., Lim D., Lee S., Lee J. H., Kim W. D., Jeong M. H., Lim J. Y., Kwon I. K., Jung Y., Park J. K., Park S. A. (2019). Heparin coating on 3D printed poly (l-lactic acid) biodegradable cardiovascular stent via mild surface modification approach for coronary artery implantation. Chem. Eng. J..

[cit98] Lin M., Lou C., Lin J., Lin T., Chen Y., Lin J. (2018). Biodegradable polyvinyl alcohol vascular stents: structural model and mechanical and biological property evaluation. Mater. Sci. Eng., C.

[cit99] McMahon S., Bertollo N., Cearbhaill E. D. O., Salber J., Pierucci L., Duffy P., Dürig T., Bi V., Wang W. (2018). Bio-resorbable polymer stents: a review of material progress and prospects. Prog. Polym. Sci..

[cit100] Oosterbeek R. N., Zhang X., Best S. M., Cameron R. E. (2022). The evolution of the structure and mechanical properties of fully bioresorbable polymer-glass composites during degradation. Compos. Sci. Technol..

[cit101] Jana P., Shyam M., Singh S., Jayaprakash V., Dev A. (2021). Biodegradable polymers in drug delivery and oral vaccination. Eur. Polym. J..

[cit102] Kellum J. A. (2000). Determinants of blood pH in health and disease. Crit. Care.

[cit103] He L., Xin J., Chen S. (2017). Robust and low cytotoxic betaine-based colorimetric pH sensors suitable for cancer cell discrimination. Sens. Actuators, B.

[cit104] Chalitangkoon J., Monvisade P. (2021). Synthesis of chitosan-based polymeric dyes as colorimetric pH-sensing materials: Potential for food and biomedical applications. Carbohydr. Polym..

[cit105] Kostka L., Kotrchová L., Šubr V., Libánská A., Ferreira C. A., Malátová I., Lee H. J., Barnhart T. E., Engle J. W., Cai W., Šírová M., Etrych T. (2020). HPMA-based star polymer biomaterials with tuneable structure and biodegradability tailored for advanced drug delivery to solid tumours. Biomaterials.

[cit106] Huang Q., Yu H., Wang L., Shen D., Chen X., Wang N. (2021). Synthesis and testing of polymer grafted mesoporous silica as glucose-responsive insulin release drug delivery systems. Eur. Polym. J..

[cit107] Ebrahimifar M., Taherimehr M. (2021). Evaluation of in-vitro drug release of polyvinylcyclohexane carbonate as a CO_2_-derived degradable polymer blended with PLA and PCL as drug carriers. J. Drug Deliv. Sci. Technol..

[cit108] Su H., Xu L., Hu X., Chen F., Li G., Yang Z., Wang L., Li H. (2021). Polymer grafted mesoporous SBA-15 material synthesized via metal-free ATRP as pH-sensitive drug carrier for quercetin. Eur. Polym. J..

[cit109] Sevostyanov M. A., Baikin A. S., Sergienko K. V., Shatova L. A., Kirsankin A. A., Baymler I. V., Shkirin A. V., Gudkov S. V. (2020). Biodegradable stent coatings on the basis of PLGA polymers of different molecular mass, sustaining a steady release of the thrombolityc enzyme streptokinase. React. Funct. Polym..

[cit110] Xing Y., Qiu L., Liu D., Dai S., Sheu C. L. (2023). The role of smart polymeric biomaterials in bone regeneration: a review. Front. Bioeng. Biotechnol..

